# Overall survival in *EGFR* mutated non-small-cell lung cancer patients treated with afatinib after EGFR TKI and resistant mechanisms upon disease progression

**DOI:** 10.1371/journal.pone.0182885

**Published:** 2017-08-30

**Authors:** A. J. van der Wekken, J. L. Kuiper, A. Saber, M. M. Terpstra, J. Wei, T. J. N. Hiltermann, E. Thunnissen, D. A. M. Heideman, W. Timens, E. Schuuring, K. Kok, E. F. Smit, A. van den Berg, H. J. M. Groen

**Affiliations:** 1 Department of Pulmonary Diseases, University of Groningen, University Medical Centre Groningen, Groningen, Netherlands; 2 Department of Pulmonary Diseases, VU University Medical Centre, Amsterdam, Netherlands; 3 Department of Pathology and Medical Biology, Groningen, University of Groningen, Groningen, Netherlands; 4 University of Groningen, Department of Genetics, Groningen, Netherlands; 5 Department of Pathology, VU University Medical Centre, Amsterdam, Netherlands; 6 Department of Thoracic Oncology, Netherlands Cancer Institute, Amsterdam, Netherlands; University of Nebraska Medical Center, UNITED STATES

## Abstract

**Purpose:**

To determine survival in afatinib-treated patients after treatment with first-generation EGFR tyrosine kinase inhibitors (TKIs) and to study resistance mechanisms in afatinib-resistant tumors.

**Methods:**

Characteristics and survival of patients treated with afatinib after resistance to erlotinib or gefitinib in two large Dutch centers were collected. Whole exome sequencing (WES) and pathway analysis was performed on available pre- and post-afatinib tumor biopsies and normal tissue.

**Results:**

A total of 38 patients were treated with afatinib. T790M mutations were identified in 22/29 (76%) pre-afatinib treatment tumor samples. No difference in median progression-free-survival (2.8 months (95% CI 2.3–3.3) and 2.7 months (95% CI 0.9–4.6), p = 0.55) and median overall-survival (8.8 months (95% CI 4.2–13.4) and 3.6 months (95% CI 2.3–5.0), p = 0.14) were observed in T790M+ patients compared to T790M- mutations.

Somatic mutations in *TP53*, *ADAMTS2*, *CNN2* and multiple genes in the Wnt and PI3K-AKT pathway were observed in post-afatinib tumors of six afatinib-responding and in one non-responding patient. No new *EGFR* mutations were found in the post-afatinib samples of the six responding patients. Further analyses of post-afatinib progressive tumors revealed 28 resistant specific mutations in six genes (*HLA-DRB1*, *AQP7*, *FAM198A*, *SEC31A*, *CNTLN*, and *ESX1*) in three afatinib responding patients. No known EGFR-TKI resistant-associated copy number gains were acquired in the post-afatinib samples.

**Conclusion:**

No differences in survival were observed in patients with *EGFR*-T790M treated with afatinib compared to those without T790M. Tumors from patients who had progressive disease during afatinib treatment were enriched for mutations in genes involved in Wnt and PI3K-AKT pathways.

## Introduction

Most patients with advanced non-small cell lung carcinoma (NSCLC) with epidermal growth factor receptor (EGFR) activating mutations will develop resistance after 6–9 months of treatment with first generation reversible tyrosine kinase inhibitors (TKIs) such as erlotinib or gefitinib[1,2]. The most common resistance mechanism is caused by the T790M gatekeeper mutation, and is detected in about half of the patients. Additional resistance-associated mechanisms are *MET* amplification, *HER2* mutations, transformation to small-cell lung cancer, expression of *IGFR1*, or alternative pathways to maintain PI3K/Akt signalling[3–7].

Because afatinib showed effectiveness in erlotinib resistant lung cancer models, afatinib effectiveness was studied in the Lux-Lung 4 study[8]. In this study patients with acquired resistance to first generation EGFR-TKIs exhibited a low response rate to afatinib and consequently the study did not meet its primary endpoint[9]. Reported resistance mechanisms to afatinib after first generation TKI are other mutations in *EGFR* (e.g. V843I), *FGFR1* amplification, upregulation of IL6R/JAK1/STAT3, glycolysis and Src pathways, and autophagy[10–17].

Pooled analysis of the Lux-Lung 3 and 6 trials showed a superior overall survival (OS) for first-line afatinib of 31.7 months for exon 19del *EGFR* mutations versus 20.7 months for the chemotherapy group (HR 0.59 (95%CI 0.45–0.77); p = 0.001). In contrast, no significant effect on OS of afatinib was observed in the L858R group (22.1 months versus 26.9 months in the chemotherapy group (HR 1.25 (95%CI 0.92–1.71); p = 0.16)[18]. Direct comparison of first-line gefitinib vs. afatinib treated patients revealed a significantly improved progression free survival (PFS) for patients treated with afatinib in a phase 2b trial[19]. Treatment of *EGFR*^L858R/T790M^ mutant cell lines with rociletinib and osimertinib, targeting T790M, revealed a strong inhibition on cell growth[20]. In lung cancer patients, tumour responses with these compounds were observed in 58% and 68% of patients with T790M mutation, respectively[21,22].

The T790M mutation plays a role as mechanism of resistance after first line treatment with afatinib as well[23]. However, in an Eastern Asian study, T790M played no role in treatment outcome or the prognosis of patients treated with second-line afatinib indicating a similar effect on both T790M positive and negative tumour clones[24]. The development of late occurring T790M clones in tumours may go along with other resistant mechanisms than early developing T790M clones.

In this study we analysed survival of mostly white patients treated with afatinib after becoming resistant to erlotinib or gefitinib. In addition, we investigated the development of afatinib resistant associated mutations using whole exome sequencing (WES) in a subset of patients.

## Materials and methods

### Patient selection

Patients with relapsed advanced NSCLC whose tumour had progressed following initial disease control for more than 12 weeks with gefitinib or erlotinib and subsequently treated with afatinib 40 mg daily, partly on a compassionate use program, were enrolled, in two Dutch University Medical Centres (Free University Medical Centre and University Medical Centre Groningen)[25]. Patient characteristics including number of treatment lines, duration of previous EGFR-TKI exposure, the duration of afatinib use and PFS and OS were recorded. Informed consent for tumour tissue from all patients was obtained before biobanking and retrieval from the Groningen Pathology biobank and VUMC Pathology biobank. All patient data were anonymised and de-identified prior to analysis. The authors were not informed about identification variables. The study was approved by the Medical Ethical Committee of the University Medical Centre Groningen and conducted in accordance with the provisions of the Declaration of Helsinki and Good Clinical Practice guidelines. Due to the retrospective nature of this study on biobanking material, under Dutch Law for human medical research (WMO), no specific written permission was compulsory from the Institutional Review Board.

### Tumour response measurement

Tumour responses were assessed by comparing CT of chest and abdomen before start of afatinib, and every 6 weeks during treatment using RECIST version 1.1 criteria[26]. This means that if there was more than 30% shrinkage of the tumour and metastases, this was called a partial response (PR). If more than 20% growth of the tumour was found, this was called progressive disease (PD). Otherwise we called this stable disease (SD).

### Tumour biopsies and diagnostic molecular analysis

Tumour biopsies were tested for the presence of *EGFR* mutations before and after treatment with erlotinib or gefitinib. Re-biopsies were taken for WES prior to start of afatinib and upon subsequent tumour progression. Paired blood or normal tissue was used as control to filter for personal variants. Briefly, 3-micron paraffin embedded tumour tissue sections were stained with haematoxylin and eosin and assessed for tumour content. Subsequent tissue sections of 10 micron were used for DNA isolation. Diagnostic testing for mutations was performed using high resolution melting analysis (HRM) for *EGFR* exons 18, 19, 20 and 21 (CCDS5514.1), for *KRAS* exon 2 for codon 12, 13, 61 (CCDS8702.1) and for *BRAF* exon 15 (NM_004333)[27,28]. PCR products with an abnormal HRM curve were re-amplified and subjected to Sanger sequencing to identify the mutation. *ALK* and *ROS1* translocations were determined by Abbott FISH tests (Abbott 06N38-020 and Abbott 08N29-020), respectively.

### Whole exome sequencing

In cases of tumour content less than 50%, laser microdissection (LMD6000, Leica, Wetzlar, Germany) was used. DNA from FFPE samples for WES was isolated using ReliaPrep^™^ FFPE gDNAMiniprep System kit (Promega, Madison, USA) following the protocol of the manufacturer. A standard salt-chloroform protocol was used to isolate DNA from blood. Quality control and WES were performed by BGI (BGI Tech Solutions Co. Ltd, Hong Kong). Raw image files were processed by Illumina base-calling Software 1.7 for base-calling with default parameters (Illumina Inc., San Diego, USA).

Reads were aligned to the human 1000 genomes reference based on the GRCh37 build using BWA 5.9rc[29]. Picard tools were used for format conversion and marking duplicate reads. Genome Analysis Toolkit (GATK) was used for indel realignment and base score quality recalibration (BSQR) by Molgenis Compute 4[30,31]. After using custom scripts in the VCF tools library, variant calling was performed using the GATK unified genotype and variant annotation by using SNPEFF/SNPSIFT 3.5 with the ensembl release 74 gene annotations http://www.ensembl.org/index.html), dbNSFP2.3, and GATK with annotations from the Database of Single Nucleotide Polymorphisms (dbSNP) Bethesda (MD): National Centre for Biotechnology Information, National Library of Medicine (dbSNP Build ID: 137) and CosmicCodingMuts_v62[32–35]. For mutations with a moderate impact according to SNPEFF, we used the CADD value to discriminate between mutations with a possible (CADD score >10) or a probable effect (CADD >20) on protein function. Exome sequencing data have been deposited on European Nucleotide Archive (ENA) website and are available under accession number: PRJEB21459 (http://www.ebi.ac.uk/ena/data/view/PRJEB21459).

### Identification of afatinib resistance associated mutations

Different criteria were used to identify mutations associated with resistance to afatinib treatment. First, we eliminated variants with a total read count of less than 10 in corresponding normal DNA, as we were not able to exclude them as personal variants (step 1). Then, we excluded germline variants based on mutant read count of more than one and a total read count of 10–49, or mutant read count of more than two and a total read count of ≥50 in the normal DNA (steps 2 and 3). The remaining variants were regarded as true somatic mutations. Next, we filtered out variants with less than 10x coverage in either primary or resistant biopsies (step 4), as read counts for these variants are too low to be used for identification of afatinib resistance associated mutations.

As we did not have pre-afatinib tumour sample for all seven patients, that also had post-afatinib samples, we followed two different strategies to identify potential resistance-related mutations: a) for all seven patients with adequate tumour samples we generated a list of genes having a mutation in the resistant sample irrespective of having a pre-afatinib sample or not, b) for 3 out of 7 patients with both pre- and post-afatinib samples, we selected variants with a more than two times higher mutant read frequency (MRF) in the resistant versus the primary biopsy (MRF_R_>2*MRF_P_; step 5).

In the final step of both analyses, we only included variants with a mapping quality >20 and a quality score >20. Genes found in this analysis were browsed in the Exome Aggregation Consortium (ExAC), Cambridge, MA (URL: http://exac.broadinstitute.org) [accessed JUL-2016] to screen for any remaining known single nucleotide variants (SNVs). The COSMIC database was used to compare identified mutations in our cohort to the reported somatic mutations in cancer (http://cancer.sanger.ac.uk/cosmic) [accessed AUG-2016].

### Pathway analysis

Partek Genomics Suite 6.6 (Partek Inc., St Louis, MO) was used to link mutated genes to either particular pathways only or whether they belonged to the same pathways.

### WES-based copy number variant analysis (CNV)

Pseudo probe data were generated with VarScan2 and Samtools as described previously by Koboldt et al. and Li et al.[36,37]. Briefly, for each sample the pseudo probe derived GC-normalized log2 copy number ratios were generated by dividing the read counts of the tumour sample by the read counts of the corresponding normal sample. All alignments with a mapping quality greater than 40 in combination with a minimal segment size of 2kb and a maximal segment size of 5kb with a mean coverage of at least one were used to calculate the log2 ratios.

CNV plots of the post-afatinib tumour were compared to the CNV plot of the pre-afatinib tumour of the same patient by a combination of calculated ratios and visual inspection.

### Statistical analysis

Descriptive statistics were used for the patient characteristics. Objective tumour response rate (ORR) was defined as the best response to treatment of complete response (CR) or partial response (PR) according to RECIST 1.1[26]. PFS was defined as the time from start of first generation TKI or start of afatinib in calculating PFS of erlotinib and gefitinib or afatinib treatment, respectively, until progressive disease (PD) according to RECIST 1.1 or death and OS was defined as the time from start of these treatments until death or lost to follow up. Patients who had not progressed at data cut-off were censored at the last day of follow-up. PFS and OS were estimated with Kaplan-Meier survival curves using log-rank test for estimating group differences. Chi-square Test was used to compare group variables. P-values <0.05 were considered significant. Statistical analyses were performed with SPSS-Statistics version 22.0 (IBM corporation, Armonk, NY, USA).

## Results

### Study population

**Fig 1:**
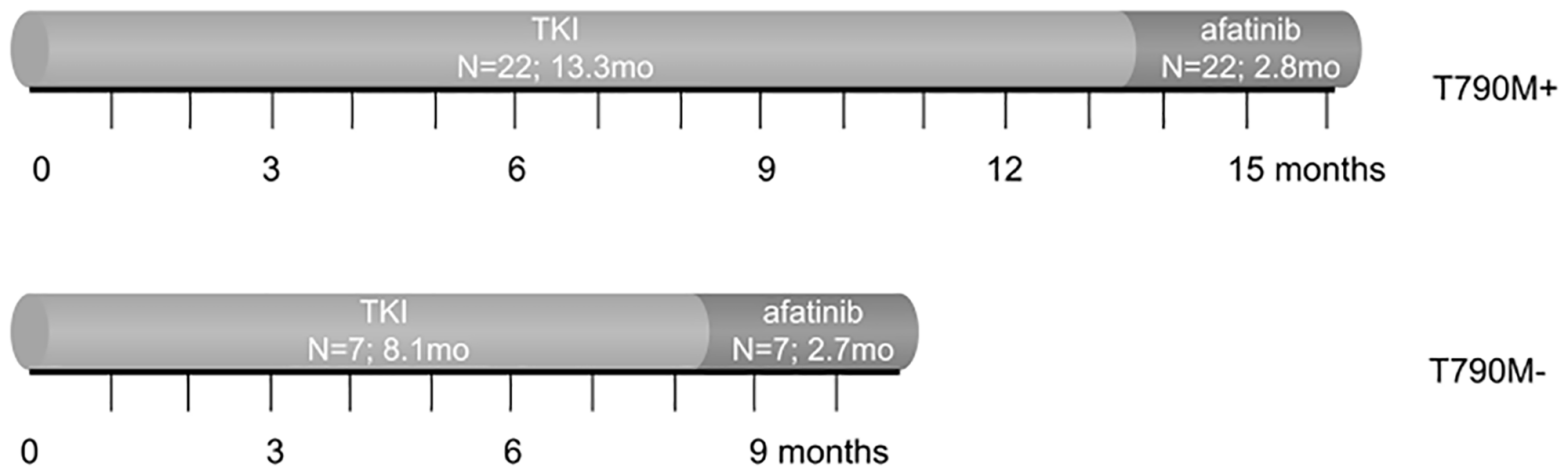
Median PFS for sequential treatments in T790M positive and negative NSCLC patients. After first generation EGFR-TKI, 22 patients had a T790M and 7 did not. All patients received afatinib, afterwards.

Between April 2009 and January 2014, 38 patients with advanced adenocarcinoma of the lung, from two Dutch university hospitals, were treated with afatinib (S1 Fig). Follow-up was more than 18 months after the last patient was included. All patients received gefitinib or erlotinib prior to afatinib, two patients received erlotinib, followed by gefitinib. A platinum doublet was given as first line treatment to 24 patients before treatment with first generation TKI and afatinib (Fig 1).

### Efficacy of 1^st^ line and 2^nd^ line TKI treatments

Median PFS on first-line erlotinib or gefitinib TKI treatment in those who turned out to be T790M positive (n = 22) and negative (n = 7) in later biopsies showed a trend to be different, 13.3 months (95% CI., 10–17) and 8.1 months (95% CI., 0–16) respectively (p = 0.06; Fig 1).

Tumour response rate of all 38 patients on second line afatinib was 18% and the disease control rate was 79%. Median PFS on afatinib was 2.8 months (95% CI., 2.3–3.2) and median OS was 6.9 months (95% CI., 1.5–12.4).

### Survival by mutation type in afatinib treated patients

**Fig 2:**
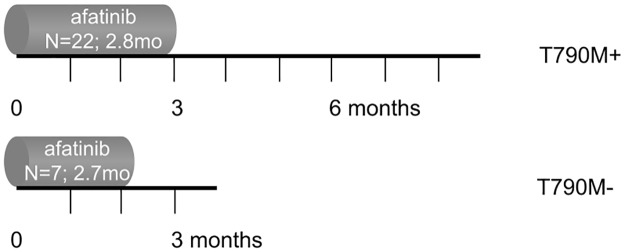
Median PFS in OS axis for sequential treatments in T790M positive and negative advanced NSCLC patients. Survival outcome of afatinib treatment is shown after first generation EGFR-TKI treatment. The X-axis represents the overall survival. The bars indicate the progression free survival for afatinib.

Median PFS of afatinib treated patients with (n = 22) and without (n = 7) T790M mutation was similar with 2.8 months (95% CI 2.3–3.3) and 2.7 months (95% CI 0.9–4.6), respectively (p = 0.55; Fig 1). Median OS was numerical better in the T790M positive as compared to the T790M negative group, although not significant (8.8 months (95% CI, 4.2–13.4) and 3.6 months (95% CI, 2.3–5.0); p = 0.14; Fig 2).

### EGFR mutational analysis

The first biopsy was taken before start of any TKI treatment (n = 38). Thirty-three patients had activating *EGFR* mutations, the most common mutation was a deletion in exon 19 (87%; n = 24) (Table 1); no T790M mutations were observed in any of the biopsies taken before EGFR-TKI. Four patients with wild type *EGFR* and one patient with no test result available were included based on Jackman’s criteria (TKI treatment with at least stable disease for 6 months)[25]. None of those five patients had *KRAS* or *BRAF* mutations or *ALK* and *ROS1* translocations.

**Table 1:** EGFR mutation status in tumor biopsies of a cohort of 38 advanced NSCLC patients.

EGFR mutation	First biopsy (n = 38)	Pre-afatinib biopsy (n = 33)	Post-afatinib biopsy (N = 18)	WES (N = 7)
Exon 18	1	0	1	0
Exon 19	24	4	5	2
Exon 19 + T790M		18	6	1
WT				1
Exon 21	7	2	2	0
Exon 21 + T790M		4	2	2
Exon 18 + 20	1	1	0	0
WT	4	4	2	0
Exon 18 + 21 +T790M				1
Mutation analysis not possible	1	0	0	0

A diagnostic biopsy taken before erlotinib and/or gefitinib treatment is called first biopsy; A biopsy after first generation EGFR-TKI is called pre-afatinib biopsy. Biopsy taken in patients who responded to afatinib and underwent a biopsy afterwards is called post-afatinib biopsy. WT: wild type.

A second biopsy taken after failure on erlotinib or gefitinib was available of twenty-nine *EGFR*+ patients. In 22/29 (76%) patients with a known activating *EGFR* mutation the T790M mutation was detected as a second mutation. The presence of a T790M mutation was significantly more common in patients treated with erlotinib alone (18/19; 95%) compared to patients treated with gefitinib alone (2/8; 25%, p<0.001; Table 2). The two patients treated with erlotinib and gefitinib were excluded from this comparison. No cancer cell transformation has been observed in our population.

**Table 2:** Patient characteristics of the afatinib treated group according to T790M mutation.

	T790M + (N = 22)	T790M − (N = 7)	P-value
Median age (years; range)	60 (32–81)	56 (44–67)	0.45
Sex			0.55
Male	4	2	
Female	18	5	
ECOG Performance status			0.12
0	12	1	
1	9	6	
2	1	0	
Ethnic origin			0.49
White	19	7	
Asian	2	0	
Afro-American	1	0	
Smoking history (at start afatinib treatment)			0.33
Never smoker	10	5	
Ex-smoker	10	1	
Current smoker	0	0	
Unknown	2	1	
Number of lines of previous chemotherapy			0.29
0	11	1	
1	3	4	
2	6	2	
>2	2	0	
EGFR TKI before afatinib			<0.001
erlotinib	18	1	
gefitinib	2	6	
both subsequently	4	1	

### Whole exome sequencing (WES)

Out of the 18 patients with a post-afatinib biopsy, there was enough tumour tissue to perform WES in six patients. Normal, pre- and post-afatinib samples were available in 3 of the 6 responding patients (#2, 5 and 6) and only normal and post-afatinib samples with sufficient DNA quality were available from the other three patients (#1, 4 and 7). In only one (patient #3) out of nine non-responders to afatinib, there was enough tumour tissue in the pre-afatinib biopsy. Of the non-responding patient we analysed pre-afatinib normal and tumour tissue samples.

In the initial analyses we focused on recurrently mutated genes found in at least 2 of the 7 biopsies irrespective of presence of the mutation in the pre-treatment biopsy. This revealed presence of 284 mutations in 68 genes (S1 Table). According to putative damaging effect according to CADD a high score (>20) was found for 27 mutations in 25 genes, e.g. *TP53*, *DYNC2H1*, *MGA*, *USH2A*, *ROBO2*, *TEX15*, *ADAMTS2*, *CUL7*, *IL33*, *ADAMTS12*, *CNN2*, *CNKSR3*, *LAMA3*, *EML6*, *TTN*, *KNDC1*, *EPB41*, *PKHD1L1*, *KLHL23*, *EVPL*, *CACNA2D1*, *CDC27*, *KCNT2*, *ASTN2 and MROH2B*. A moderate CADD score (10–20) and/or recurrent mutations were found for 110 mutations in 45 genes, e.g., *OR8U1*, *MUC16*, *MUC6*, *ANKRD36C*, *HLA-DRB5*, *DYNC2H1*, *NEFH*, *FASLG*, *MUC5B*, *PRSS3*, *HYDIN*, *MGA*, *USH2A*, *TAS2R43*, *GRP98*, *C4BPB*, *DOCK2*, *INVS*, *CUL7*, *NHSL2*, *USP24*, *TPSB2*, *MUC12*, *OR2T4*, *CNN2*, *CNKSR3*, *LAMA3*, *TTN*, *KNDC1*, *EPB41*, *EVPL*, *FZR1*, *CACNA2D1*, *CDC27*, *KCNT2*, *EMP2*, *MST1*, *ARHGAP32*, *NLGN4X*, *HLADQA1*, *HERC2*, *ASTN2*, *SP8*, *PRSS1 and MROH2B*. *CNN2* is the only gene with an identical high CADD score mutation in 2 patients. Some of the other recurrently mutated genes had mixed high and moderate CADD scores. In total, 137 mutations in 48 genes were identified as potentially involved in afatinib resistance.

In patient #7 treated with afatinib according to Jackman’s criteria, without a known *EGFR* mutation, WES revealed activation mutations in *EGFR*, e.g. G719C and a L861Q. In the other six patients, no new *EGFR* mutations were identified. For the three pre-afatinib samples this was consistent with the targeted diagnostic mutation tests.

In patients #2, 5, and 6 (Table 3) WES data of normal tissue, pre- and post-afatinib tumour samples could be analysed.

**Table 3:** Recurrently mutated genes in tumors from patients who progressed under afatinib.

GENE	PATIENT	CHROM	POS	REF	ALT	AA CHANGE	CADD score
HLA-DRB1	#2	6	32552130	C	A	R42S	0.145
			32552131	C	G	R42T	0.005
	#6		32552134	T	G	K41T	10.93
	#3		32552130	C	A	R42S	0.145
			32552132	T	A	R42W	10.52
	#4		32548544	T	G	I248L	16.04
	#7		32552130	C	A	R42S	0.145
			32552131	C	G	R42T	0.005
			32552137	G	C	P40R	0
			32552138	G	C	P40A	0.005
			32552143	C	T	W38*	13.84
			32552144	A	C	W38G	3.518
AQP7	#2	9	33385808	G	T	N194K	15.08
	#5		33385709	C	T	V96I	9.787
			33385712	G	A	P95S	10.22
			33385690	C	T	R234S	14.5
			33385698	A	G	L231P	16.61
	#1		33385712	G	A	P95S	10.22
	#4		33395131	TG	T	Q30	NA
FAM198A	#2	3	43074734	C	A	P327T	9.704
	#6		43074337	G	T	W194C	14.09
SEC31A	#5	4	83803067	C	T	R8H	27.8
	#6		83784534	CT	C	E482	NA
CNTLN	#2	9	17366624	TGAA	T	E633	NA
	#5		17236574	TG	T	A280	NA
			17236576	C	A	A280E	5.077
ESX1	#2	X	103497493	G	C	R175G	11.68
	#6		103498982	C	A	G120V	14.79

Tumor samples were from 3 patients (#2, #5 and #6) with WES data of normal, primary and resistant tumors and from patients with samples from resistant tumors only.

chrom, chromosome; pos, basepair location; ref/alt, reference and altered nucleotides at mutated position; AA change, amino acid change; CADD, Combined Annotation Dependent Depletion score; CADD score ≥10 indicates a position within the top 10% most deleterious mutations. A score of ≥20 indicates a position within the top 1% most deleterious mutations.

Four hundred forty five mutations in 367 genes (range 87–216) had higher MRF, or were specific, for the post-afatinib treatment sample. Mutations in these genes might have contributed to the observed afatinib resistance. Six genes, with in total 28 mutations, were recurrently mutated in at least two out of three patients, i.e. *HLA-DRB1*, *AQP7*, *FAM198A*, *SEC31A*, *CNTLN*, and *ESX1*.

Most of the mutations observed in *HLA-DRB1* were also present in the ExAc database and therefore appear to be less important. The other mutations, absent in the ExAc database but present in the COSMIC database (in different tumour types), might be relevant, such as p.W38fs* in *HLA-DRB1*, p.Q30fs* in *AQP7*, p.C135R in *TP53*, p.Q220* in *HLA-DRB5*, p.G8V in *PRSS3*, p.S1155R in *USH2A* and p.V521I in *KCNT2*. The p.K41T mutation in *HLA-DRB1*, p.IT255T and p.Q136E in *TP53* and p.P2811S in *USH2A* are also described in human lung cancer samples. The p.P95S mutation in *AQP7* was observed in patients #1 and #5.

Pathway analysis of all genes mutated in any of the 7 analysed patients indicated that most of the mutated genes were members of Wnt signalling (S2 Fig) and PI3K-AKT (S3 Fig) pathways. In addition, we observed mutations in two genes of the glycolysis pathway (Table 4). We did not identify mutations in genes related to the pathways known to be associated with afatinib resistance, e.g. autophagy and IL6R/JAK/STAT.

**Table 4:** Overview of significantly involved pathways in patients’ progressive disease on afatinib and the involved mutated genes.

Nr. of patient sample	N/P/R						N/R			N/P
	#2		#5		#6		#1	#4	#7	#3
	A	B	A	B	A	B	C	C	C	D
**Pathway**										
Glycolysis					ADPGK			LDH6A		
Autophagy										
IL6/JAK/STAT										
Wnt	MAPK8	TP53		TP53	FOSL1	PLCB4	WNT9B	INVS	AXIN1	*TP53*
		LEF1					*TP53*		*TP53*	
							FZD10			
							APC2			
PI3K-AKT	COL4A4	ITGA2	PPP2R2B	EGFR	LAMC3	EGF	*EGFR*	*EGFR*	*TP53*	*KDR*
		EGFR		PIK3CA		EGFR	LAMB3	FASLG		RELN
		VWF		PIK3CG		COL1A2	PTEN			FASLG
		TSC2		COL4A5			FASLG			*TP53*
		TP53					*TP53*			

A: resistant biopsy specific based on MRF R >2x MRF P, B: mutation present in both pre and post afatinib biopsy based on MRF R <2x MRF P and MRF R > 0. C: mutation in resistant biopsy based on MRF R > 0, D: mutation in pre-afatinib biopsy in non-responding patient based on MRF P > 0; *italic*: present in other primary samples. N: normal tissue or buffy coat, P: biopsy before afatinib treatment, R: biopsy after afatinib treatment.

### Copy number variations

We observed only a few changes in copy number variations (CNV) between the pre- and post-afatinib samples. In patient #2 copy number gain (CNG) of part of chromosome 5, 8, 11 and 16 and loss of part of chromosome 4 and 14 was observed (S4A Fig). In patient #5 no differences in CNV between pre- and post-afatinib biopsies was observed (S4B Fig). In patient #6, copy number loss was seen only for part of chromosome X (S4C Fig). Specifically, no CNV aberrations in *MET*, *FGFR1*, Src or genes involved in the IL6/JAK1/STAT3 pathway were found.

## Discussion

In this study we investigated afatinib resistance in patients with relapsed advanced NSCLC whose tumour had progressed on gefitinib or erlotinib and subsequently were treated with afatinib. In 38 patients with an *EGFR* mutation or treated with TKIs according to Jackman’s criteria we first determined the relevance of erlotinib and gefitinib in inducting T790M mutation in *EGFR*. The exon 20 T790M mutation was detected under EGFR-TKI selection pressure in re-biopsies of 22/29 (76%) patients in our cohort. This percentage is slightly above the upper range that has been reported in the literature (25–63%)[38–40]. The percentage of T790M+ patients was significantly higher in the erlotinib treated as compared to the gefitinib treated patients. In the literature there is a trend that T790M mutations are numerically higher in patients who received erlotinib[41]. The duration of first line reversible TKIs did not influence the occurrence of a T790M mutation. In our cohort of afatinib treated patients, PFS (2.8 months) and OS (9.2 months) were similar to the Lux-Lung 1 study[42]. We did not find an influence of the pre-afatinib treatment induced T790M mutation on response outcome (PFS or OS) on second line afatinib treatment. This is consistent with the study of Sun et al. on an Asian population. In contrast Landi et al. found no tumour response with afatinib in T790M positive patients. In the current literature discrepancy is observed in the tumour response to afatinib in T790M positive patients[24,43].

To understand molecular events underlying progression of disease on afatinib treatment, WES was performed in all patients with sufficient tumour tissue to identify known and novel resistance mechanisms. We observed 68 recurrently mutated genes in 7 different patients with progression under afatinib, of which 137 mutations in 48 genes are probably involved in afatinib resistance based on moderate or high CADD score. The R287Q mutation in *CNN2* is noteworthy based on the high CADD score and being identified in two patients. *CNN2* has been described in prostate cancer and is involved in cell migration and cell morphology[44]. This gene is probably involved in rectal cancer as well[45]. Post-afatinib specific mutations were observed in *HLA-DRB1*, *AQP7*, *FAM198A*, *SEC31A*, *CNTLN* and *ESX1*.

The observed resistance associated mutations were present in a broad range of genes. Therefore, we explored if these genes clustered in specific pathways that might play a role in the progression of disease while being on afatinib treatment. We found that most of the genes with mutations were part of the Wnt and/or PI3K-AKT pathways. Mutations in genes related to the Wnt pathway were also implicated in erlotinib resistance in *EGFR* mutation positive lung cancer cell lines[46,47]. In gastric cancer for example, a role for Wnt signalling has been observed to influence disease behaviour[48].

Inhibiting the Wnt pathway is an attractive treatment option for patients with resistant cancers and is now tested in phase I trials. The PI3K-AKT pathway has not been associated with afatinib resistance previously, except perhaps the hint observed in gefitinib resistant NSCLC patients treated with Paris Saponins which induced apoptosis via the PI3K-AKT pathway in the tumour cells[49]. In melanoma, for example, AKT signalling is an important resistant mechanism in BRAF positive cancer cells [50].

CNVs of different genes have been associated with resistance in *EGFR* mutant lung cancer[51]. However, in our cohort of afatinib resistant patients, no known specific afatinib resistance-related CNVs were observed. Together with the WES data, this suggests that in our patients, mutations in *EGFR* or *IGFR1*, genomic aberrations in *MET*, *FGFR1* amplification, mutations in *SRC* or in the IL6R/JAK1/STAT3 pathway, previously reported as resistance mechanisms by association in few patients or in cell lines, were not observed in our study to be involved in afatinib resistance[5,10].

In conclusion, no differences in survival were observed in patients with *EGFR* T790M treated with afatinib compared to those without T790M. Potential mechanism of resistance to afatinib treatment might be related to mutations in *HLA-DRB1*, *AQP7*, *TP53*, *HLA-DRB5*, *PRSS3*, *USH2A*, *KCNT2 and CNN2* and to mutations in genes of the Wnt and PI3K-AKT pathways.

## Supporting information

S1 Table Recurrently mutated genes in tumor samples from patients resistant to afatinib without information from primary biopsies using whole exome sequencing chrom, chromosome; pos, bp location; ref/alt, reference and altered nucleotides at mutated position; AA change, amino acid change; CADD, Combined Annotation Dependent Depletion score; CADD score ≥10 indicates a position within the top 10% most deleterious mutations. A score of ≥20 indicates a position within the top 1% most deleterious mutations.
(DOCX)

S1 Fig Overview of treatment in 38 patients administered per treatment line. Organogram of 38 treated patients where afatinib is given in different treatment lines (1–5). Chemotherapy was variable, e.g. cisplatinum/pemetrexed, carboplatinum/paclitaxel/bevacizumab, docetaxel, pemetrexed.
(TIF)

S2 Fig Wnt signaling pathway involved in afatinib resistant tumours. Color boxes are the different mutations found in different samples. Multiple mutated genes involved in the Wnt pathway are shown.
(TIF)

S3 Fig PIK3-AKT signaling pathway involved in afatinib resistant tumours. Color boxes are the different mutations found in different samples.
(TIF)

S4 Fig Overview of copy numbers and their aberrations in patients #2, #5 and #6. Overview of the copy numbers and allele frequencies of pre-afatinib (top) and post-afatinib biopsies (bottom) in patients 2 (A), 5 (B) and 6 (C). The boxes indicate aberrations between pre-afatinib and post-afatinib biopsies with either copy number gain or loss.
(TIF)

## References

[1] Rosell R, Carcereny E, Gervais R, Vergnenegre A, Massuti B, Felip E, et al Erlotinib versus standard chemotherapy as first-line treatment for European patients with advanced EGFR mutation-positive non-small-cell lung cancer (EURTAC): a multicentre, open-label, randomised phase 3 trial. Lancet Oncol. 2012;13: 239–246. doi: 10.1016/S1470-2045(11)70393-X 22285168 10.1016/S1470-2045(11)70393-X

[2] Mok TS, Wu YL, Thongprasert S, Yang CH, Chu DT, Saijo N, et al Gefitinib or Carboplatin-Paclitaxel in Pulmonary Adenocarcinoma. N Engl J Med. 2009;361: 947–957. doi: 10.1056/NEJMoa0810699 19692680 10.1056/NEJMoa0810699

[3] Engelman JA, Janne PA. Mechanisms of acquired resistance to epidermal growth factor receptor tyrosine kinase inhibitors in non-small cell lung cancer. Clin Cancer Res. 2008;14: 2895–2899. doi: 10.1158/1078-0432.CCR-07-2248 18483355 10.1158/1078-0432.CCR-07-2248

[4] Ayoola A, Barochia A, Belani K, Belani CP. Primary and acquired resistance to epidermal growth factor receptor tyrosine kinase inhibitors in non-small cell lung cancer: an update. Cancer Invest. 2012;30: 433–446. doi: 10.3109/07357907.2012.666691 22571344 10.3109/07357907.2012.666691

[5] Yeo CD, Park KH, Park CK, Lee SH, Kim SJ, Yoon HK, et al Expression of insulin-like growth factor 1 receptor (IGF-1R) predicts poor responses to epidermal growth factor receptor (EGFR) tyrosine kinase inhibitors in non-small cell lung cancer patients harboring activating EGFR mutations. Lung Cancer. 2015;87: 311–317. doi: 10.1016/j.lungcan.2015.01.004 25617986 10.1016/j.lungcan.2015.01.004

[6] Takezawa K, Pirazzoli V, Arcila ME, Nebhan CA, Song X, de Stanchina E, et al HER2 amplification: a potential mechanism of acquired resistance to EGFR inhibition in EGFR-mutant lung cancers that lack the second-site EGFRT790M mutation. Cancer Discov. 2012;2: 922–933. doi: 10.1158/2159-8290.CD-12-0108 22956644 10.1158/2159-8290.CD-12-0108 PMC3473100

[7] Tanizaki J, Okamoto I, Okamoto K, Takezawa K, Kuwata K, Yamaguchi H, et al MET tyrosine kinase inhibitor crizotinib (PF-02341066) shows differential antitumor effects in non-small cell lung cancer according to MET alterations. J Thorac Oncol. 2011;6: 1624–1631. doi: 10.1097/JTO.0b013e31822591e9 21716144 10.1097/JTO.0b013e31822591e9

[8] Li D, Ambrogio L, Shimamura T, Kubo S, Takahashi M, Chirieac LR, et al BIBW2992, an irreversible EGFR/HER2 inhibitor highly effective in preclinical lung cancer models. Oncogene. 2008;27: 4702–4711. doi: 10.1038/onc.2008.109 18408761 10.1038/onc.2008.109 PMC2748240

[9] Katakami N, Atagi S, Goto K, Hida T, Horai T, Inoue A, et al LUX-Lung 4: a phase II trial of afatinib in patients with advanced non-small-cell lung cancer who progressed during prior treatment with erlotinib, gefitinib, or both. J Clin Oncol. 2013;31: 3335–3341. doi: 10.1200/JCO.2012.45.0981 23816963 10.1200/JCO.2012.45.0981

[10] van der Wekken AJ, Saber A, Hiltermann TJ, Kok K, van den Berg A, Groen HJ. Resistance mechanisms after tyrosine kinase inhibitors afatinib and crizotinib in non-small cell lung cancer, a review of the literature. Crit Rev Oncol Hematol. 2016;100: 107–116. doi: 10.1016/j.critrevonc.2016.01.024 26852079 10.1016/j.critrevonc.2016.01.024

[11] Saber A, van der Wekken A, Hiltermann TJ, Kok K, Van den Berg A, Groen H. Genomic aberrations guiding treatment of non-small cell lung cancer patients. Cancer Treatment Communications. 2015;4: 23–33.

[12] Matsushima S, Ohtsuka K, Ohnishi H, Fujiwara M, Nakamura H, Morii T, et al V843I, a lung cancer predisposing EGFR mutation, is responsible for resistance to EGFR tyrosine kinase inhibitors. J Thorac Oncol. 2014;9: 1377–1384. doi: 10.1097/JTO.0000000000000241 25057940 10.1097/JTO.0000000000000241

[13] Azuma K, Kawahara A, Sonoda K, Nakashima K, Tashiro K, Watari K, et al FGFR1 activation is an escape mechanism in human lung cancer cells resistant to afatinib, a pan-EGFR family kinase inhibitor. Oncotarget. 2014;5: 5908–5919. doi: 10.18632/oncotarget.1866 25115383 10.18632/oncotarget.1866 PMC4171601

[14] Kim SM, Kwon OJ, Hong YK, Kim JH, Solca F, Ha SJ, et al Activation of IL-6R/JAK1/STAT3 Signaling Induces De Novo Resistance to Irreversible EGFR Inhibitors in Non-Small Cell Lung Cancer with T790M Resistance Mutation. Mol Cancer Ther. 2012;11: 2254–2264. doi: 10.1158/1535-7163.MCT-12-0311 22891040 10.1158/1535-7163.MCT-12-0311

[15] Huang S, Benavente S, Armstrong EA, Li C, Wheeler DL, Harari PM. p53 modulates acquired resistance to EGFR inhibitors and radiation. Cancer Res. 2011;71: 7071–7079. doi: 10.1158/0008-5472.CAN-11-0128 22068033 10.1158/0008-5472.CAN-11-0128 PMC3229180

[16] Lee TG, Jeong EH, Kim SY, Kim HR, Kim CH. The combination of irreversible EGFR TKIs and SAHA induces apoptosis and autophagy-mediated cell death to overcome acquired resistance in EGFR T790M-mutated lung cancer. Int J Cancer. 2014. 10.1002/ijc.29320 25382705

[17] Takezawa K, Okamoto I, Tanizaki J, Kuwata K, Yamaguchi H, Fukuoka M, et al Enhanced anticancer effect of the combination of BIBW2992 and thymidylate synthase-targeted agents in non-small cell lung cancer with the T790M mutation of epidermal growth factor receptor. Mol Cancer Ther. 2010;9: 1647–1656. doi: 10.1158/1535-7163.MCT-09-1009 20530710 10.1158/1535-7163.MCT-09-1009

[18] Yang JC, Wu YL, Schuler M, Sebastian M, Popat S, Yamamoto N, et al Afatinib versus cisplatin-based chemotherapy for EGFR mutation-positive lung adenocarcinoma (LUX-Lung 3 and LUX-Lung 6): analysis of overall survival data from two randomised, phase 3 trials. Lancet Oncol. 2015;16: 141–151. doi: 10.1016/S1470-2045(14)71173-8 25589191 10.1016/S1470-2045(14)71173-8

[19] Park K, Tan EH, O'Byrne K, Zhang L, Boyer M, Mok T, et al Afatinib versus gefitinib as first-line treatment of patients with EGFR mutation-positive non-small-cell lung cancer (LUX-Lung 7): a phase 2B, open-label, randomised controlled trial. Lancet Oncol. 2016;17: 577–589. doi: 10.1016/S1470-2045(16)30033-X 27083334 10.1016/S1470-2045(16)30033-X

[20] Tjin Tham Sjin R, Lee K, Walter AO, Dubrovskiy A, Sheets M, Martin TS, et al In Vitro and In Vivo Characterization of Irreversible Mutant-Selective EGFR Inhibitors That Are Wild-Type Sparing. Mol Cancer Ther. 2014;13: 1468–1479. doi: 10.1158/1535-7163.MCT-13-0966 24723450 10.1158/1535-7163.MCT-13-0966

[21] Janne PA, Ramalingam SS, Yang JC, Ahn MJ, Kim S, Planchard D, et al Clinical activity of the mutatant-selective EGFR inhibitor AZD9291 in patients (pts) with EGFR inhibitor-resistant non-small cell lung cancer (NSCLC). J Clin Oncol. 2014;32:5s.

[22] Sequist LV, Soria JC, Gadgeel SM, Wakelee HA, Camidge DR, Varga A, et al First-in-human evaluation of CO-1686, an irreversible, highly selective tyrosine kinase inhibitor of mutations of EGFR (activating and T790M). 2014;ASCO.

[23] Wu SG, Liu YN, Tsai MF, Chang YL, Yu CJ, Yang PC, et al The mechanism of acquired resistance to irreversible EGFR tyrosine kinase inhibitor-afatinib in lung adenocarcinoma patients. Oncotarget. 2016;7: 12404–12413. doi: 10.18632/oncotarget.7189 26862733 10.18632/oncotarget.7189 PMC4914294

[24] Sun JM, Ahn MJ, Choi YL, Ahn JS, Park K. Clinical implications of T790M mutation in patients with acquired resistance to EGFR tyrosine kinase inhibitors. Lung Cancer. 2013;82: 294–298. doi: 10.1016/j.lungcan.2013.08.023 24035188 10.1016/j.lungcan.2013.08.023

[25] Jackman D, Pao W, Riely GJ, Engelman JA, Kris MG, Janne PA, et al Clinical definition of acquired resistance to epidermal growth factor receptor tyrosine kinase inhibitors in non-small-cell lung cancer. J Clin Oncol. 2010;28: 357–360. doi: 10.1200/JCO.2009.24.7049 19949011 10.1200/JCO.2009.24.7049 PMC3870288

[26] Eisenhauer EA, Therasse P, Bogaerts J, Schwartz LH, Sargent D, Ford R, et al New response evaluation criteria in solid tumours: Revised RECIST guideline (version 1.1). European Journal of Cancer. 2009;45: 228–247. doi: 10.1016/j.ejca.2008.10.026 19097774 10.1016/j.ejca.2008.10.026

[27] Heideman DA, Thunnissen FB, Doeleman M, Kramer D, Verheul HM, Smit EF, et al A panel of high resolution melting (HRM) technology-based assays with direct sequencing possibility for effective mutation screening of EGFR and K-ras genes. Cell Oncol. 2009;31: 329–333. doi: 10.3233/CLO-2009-0489 19759413 10.3233/CLO-2009-0489 PMC4619054

[28] Heideman DA, Lurkin I, Doeleman M, Smit EF, Verheul HM, Meijer GA, et al KRAS and BRAF mutation analysis in routine molecular diagnostics: comparison of three testing methods on formalin-fixed, paraffin-embedded tumor-derived DNA. J Mol Diagn. 2012;14: 247–255. doi: 10.1016/j.jmoldx.2012.01.011 22425762 10.1016/j.jmoldx.2012.01.011

[29] Li H, Durbin R. Fast and accurate long-read alignment with Burrows-Wheeler transform. Bioinformatics. 2010;26: 589–595. doi: 10.1093/bioinformatics/btp698 20080505 10.1093/bioinformatics/btp698 PMC2828108

[30] Boomsma DI, Wijmenga C, Slagboom EP, Swertz MA, Karssen LC, Abdellaoui A, et al The Genome of the Netherlands: design, and project goals. Eur J Hum Genet. 2014;22: 221–227. doi: 10.1038/ejhg.2013.118 23714750 10.1038/ejhg.2013.118 PMC3895638

[31] McKenna A, Hanna M, Banks E, Sivachenko A, Cibulskis K, Kernytsky A, et al The Genome Analysis Toolkit: a MapReduce framework for analyzing next-generation DNA sequencing data. Genome Res. 2010;20: 1297–1303. doi: 10.1101/gr.107524.110 20644199 10.1101/gr.107524.110 PMC2928508

[32] Danecek P, Auton A, Abecasis G, Albers CA, Banks E, DePristo MA, et al The variant call format and VCFtools. Bioinformatics. 2011;27: 2156–2158. doi: 10.1093/bioinformatics/btr330 21653522 10.1093/bioinformatics/btr330 PMC3137218

[33] Cingolani P, Platts A, Wang le L, Coon M, Nguyen T, Wang L, et al A program for annotating and predicting the effects of single nucleotide polymorphisms, SnpEff: SNPs in the genome of Drosophila melanogaster strain w1118; iso-2; iso-3. Fly (Austin). 2012;6: 80–92. 22728672 10.4161/fly.19695 PMC3679285

[34] Liu X, Jian X, Boerwinkle E. dbNSFP v2.0: a database of human non-synonymous SNVs and their functional predictions and annotations. Hum Mutat. 2013;34: E2393–402. doi: 10.1002/humu.22376 23843252 10.1002/humu.22376 PMC4109890

[35] Forbes SA, Beare D, Gunasekaran P, Leung K, Bindal N, Boutselakis H, et al COSMIC: exploring the world's knowledge of somatic mutations in human cancer. Nucleic Acids Res. 2015;43: D805–11. doi: 10.1093/nar/gku1075 25355519 10.1093/nar/gku1075 PMC4383913

[36] Koboldt DC, Zhang Q, Larson DE, Shen D, McLellan MD, Lin L, et al VarScan 2: somatic mutation and copy number alteration discovery in cancer by exome sequencing. Genome Res. 2012;22: 568–576. doi: 10.1101/gr.129684.111 22300766 10.1101/gr.129684.111 PMC3290792

[37] Li J, Yang T, Wang L, Yan H, Zhang Y, Guo Y, et al Whole genome distribution and ethnic differentiation of copy number variation in Caucasian and Asian populations. PLoS One. 2009;4: e7958 doi: 10.1371/journal.pone.0007958 19956714 10.1371/journal.pone.0007958 PMC2776354

[38] Arcila ME, Oxnard GR, Nafa K, Riely GJ, Solomon SB, Zakowski MF, et al Rebiopsy of lung cancer patients with acquired resistance to EGFR inhibitors and enhanced detection of the T790M mutation using a locked nucleic acid-based assay. Clin Cancer Res. 2011;17: 1169–1180. doi: 10.1158/1078-0432.CCR-10-2277 21248300 10.1158/1078-0432.CCR-10-2277 PMC3070951

[39] Yu HA, Arcila ME, Rekhtman N, Sima CS, Zakowski MF, Pao W, et al Analysis of tumor specimens at the time of acquired resistance to EGFR-TKI therapy in 155 patients with EGFR-mutant lung cancers. Clin Cancer Res. 2013;19: 2240–2247. doi: 10.1158/1078-0432.CCR-12-2246 23470965 10.1158/1078-0432.CCR-12-2246 PMC3630270

[40] Lee Y, Lee GK, Lee YS, Zhang W, Hwang JA, Nam BH, et al Clinical outcome according to the level of preexisting epidermal growth factor receptor T790M mutation in patients with lung cancer harboring sensitive epidermal growth factor receptor mutations. Cancer. 2014;120: 2090–2098. doi: 10.1002/cncr.28711 24737599 10.1002/cncr.28711

[41] Oxnard GR, Arcila ME, Sima CS, Riely GJ, Chmielecki J, Kris MG, et al Acquired resistance to EGFR tyrosine kinase inhibitors in EGFR-mutant lung cancer: distinct natural history of patients with tumors harboring the T790M mutation. Clin Cancer Res. 2011;17: 1616–1622. doi: 10.1158/1078-0432.CCR-10-2692 21135146 10.1158/1078-0432.CCR-10-2692 PMC3060283

[42] Miller VA, Hirsh V, Cadranel J, Chen YM, Park K, Kim SW, et al Afatinib versus placebo for patients with advanced, metastatic non-small-cell lung cancer after failure of erlotinib, gefitinib, or both, and one or two lines of chemotherapy (LUX-Lung 1): a phase 2b/3 randomised trial. Lancet Oncol. 2012;13: 528–538. doi: 10.1016/S1470-2045(12)70087-6 22452896 10.1016/S1470-2045(12)70087-6

[43] Landi L, Tiseo M, Chiari R, Ricciardi S, Rossi E, Galetta D, et al Activity of the EGFR-HER2 dual inhibitor afatinib in EGFR-mutant lung cancer patients with acquired resistance to reversible EGFR tyrosine kinase inhibitors. Clin Lung Cancer. 2014;15: 411–417.e4. doi: 10.1016/j.cllc.2014.07.002 25242668 10.1016/j.cllc.2014.07.002

[44] Verone AR, Duncan K, Godoy A, Yadav N, Bakin A, Koochekpour S, et al Androgen-responsive serum response factor target genes regulate prostate cancer cell migration. Carcinogenesis. 2013;34: 1737–1746. doi: 10.1093/carcin/bgt126 23576568 10.1093/carcin/bgt126 PMC3731805

[45] Choi SY, Jang JH, Kim KR. Analysis of differentially expressed genes in human rectal carcinoma using suppression subtractive hybridization. Clin Exp Med. 2011;11: 219–226. doi: 10.1007/s10238-010-0130-5 21331762 10.1007/s10238-010-0130-5

[46] Fang X, Gu P, Zhou CC, Ren SX, Luo BF, Zeng Y, et al Effect of Wnt signaling suppression on gefitinib in non small cell lung cancer cell lines. Zhonghua Bing Li Xue Za Zhi. 2013;42: 455–459. doi: 10.3760/cma.j.issn.0529-5807.2013.07.006 24246864 10.3760/cma.j.issn.0529-5807.2013.07.006

[47] Casas-Selves M, Kim J, Zhang Z, Helfrich BA, Gao D, Porter CC, et al Tankyrase and the canonical Wnt pathway protect lung cancer cells from EGFR inhibition. Cancer Res. 2012;72: 4154–4164. doi: 10.1158/0008-5472.CAN-11-2848 22738915 10.1158/0008-5472.CAN-11-2848 PMC3673784

[48] Ooi CH, Ivanova T, Wu J, Lee M, Tan IB, Tao J, et al Oncogenic pathway combinations predict clinical prognosis in gastric cancer. PLoS Genet. 2009;5: e1000676 doi: 10.1371/journal.pgen.1000676 19798449 10.1371/journal.pgen.1000676 PMC2748685

[49] Zhu X, Jiang H, Li J, Xu J, Fei Z. Anticancer Effects of Paris Saponins by Apoptosis and PI3K/AKT Pathway in Gefitinib-Resistant Non-Small Cell Lung Cancer. Med Sci Monit. 2016;22: 1435–1441. doi: 10.12659/MSM.898558 27125283 10.12659/MSM.898558 PMC4917328

[50] Obenauf AC, Zou Y, Ji AL, Vanharanta S, Shu W, Shi H, et al Therapy-induced tumour secretomes promote resistance and tumour progression. Nature. 2015;520: 368–372. doi: 10.1038/nature14336 25807485 10.1038/nature14336 PMC4507807

[51] Jia P, Jin H, Meador CB, Xia J, Ohashi K, Liu L, et al Next-generation sequencing of paired tyrosine kinase inhibitor-sensitive and -resistant EGFR mutant lung cancer cell lines identifies spectrum of DNA changes associated with drug resistance. Genome Research. 2013;23: 1434–1445. doi: 10.1101/gr.152322.112 23733853 10.1101/gr.152322.112 PMC3759720

