# Supplementary material for: Overall survival in EGFR mutated non-small-cell lung cancer patients treated with afatinib after EGFR TKI and resistant mechanisms upon disease progression
Source: PLoS One. 2017 Aug 30;12(8):e0182885. doi: 10.1371/journal.pone.0182885 (PMC5576694; doi:10.1371/journal.pone.0182885)
Supplement: S1 Table — A score of ≥20 indicates a position within the top 1% most deleterious mutations. (DOCX) [file pone.0182885.s001.docx]

**S1 Table.** Recurrently mutated genes in tumor samples from patients resistant to afatinib without information from primary biopsies using whole exome sequencing

| GENE | PATIENT | CHROM | POS | REF | ALT | AA CHANGE | CADD score |
| --- | --- | --- | --- | --- | --- | --- | --- |
| OR8U1 | #1 | 11 | 56143987 | G | T | E296D | 0.277 |
|  | #2 |  | 56143394 | A | G | T99A | 0.45 |
|  | #3 |  | 56143556 | T | A | F153I | 8.131 |
|  |  |  | 56143562 | A | G | M155V | 0.014 |
|  |  |  | 56143370 | G | A | V91I | 0.303 |
|  | #4 |  | 56143250 | G | A | A51T | 1.371 |
|  |  |  | 56143257 | C | A | T53K | 4.977 |
|  | #7 |  | 56143103 | G | A | A2T | 12.13 |
|  |  |  | 56143108 | C | G | H3Q | 0.249 |
| EGFR | #1 | 7 | 55242464 | AGGAATTAAGAGAAGC | A | KELREA745K | NA |
|  | #2 |  | 55249071 | C | T | T790M | 30 |
|  | #3 |  | 55242464 | AGGAATTAAGAGAAGC | A | KELREA745K | NA |
|  | #4 |  | 55242465 | GGAATTAAGA | G | ELR746 | NA |
|  |  |  | 55242478 | G | C | A750P | 21.8 |
|  |  |  | 55249071 | C | T | T790M | 30 |
|  | #5 |  | 55249071 | C | T | T790M | 30 |
|  |  |  | 55259515 | T | G | L858R | 25.1 |
|  | #6 |  | 55249071 | C | T | T790M | 30 |
|  |  |  | 55259515 | T | G | L858R | 25.1 |
| TP53 | #1 | 17 | 7577514 | GTGA | G | IT255T | NA |
|  | #3 |  | 7578527 | A | G | C135R | 22.8 |
|  | #7 |  | 7578524 | G | C | Q136E | 26.1 |
| MUC16 | #1 | 19 | 9064752 | T | A | K7565I | 4.321 |
|  | #3 |  | 9002623 | C | T | R13398H | 8.006 |
|  |  |  | 9006764 | T | G | T13162P | 2.395 |
|  | #4 |  | 9002612 | T | G | K13402Q | 6.712 |
|  | #7 |  | 9008344 | A | G | S13070P | 1.811 |
|  |  |  | 9027548 | G | A | T12199I | 10.15 |
|  |  |  | 9027560 | C | T | S12195N | 6.373 |
| MUC6 | #2 | 11 | 1018408 | G | T | P1465T | 3.885 |
|  | #3 |  | 1018393 | T | C | M1470V | 1.88 |
|  | #4 |  | 1018218 | T | G | H1528P | 4.587 |
|  | #6 |  | 1016055 | G | T | P2249Q | 12.76 |
|  |  |  | 1018218 | T | G | H1528P | 4.587 |
|  | #7 |  | 1018088 | TG | T | P1571 | NA |
|  |  |  | 1018093 | G | GT | P1569P? | NA |
| ANKRD36C | #3 | 2 | 96525702 | C | T | S1268N | 0.387 |
|  | #4 |  | 96610410 | T | A | N486Y | 5.517 |
|  |  |  | 96610413 | C | T | E485K | 9.365 |
|  |  |  | 96610420 | G | T | D482E | 4.959 |
|  | #5 |  | 96619713 | C | A | E393* | 12.16 |
|  | #7 |  | 96616501 | C | CAA | V430V? | NA |
|  |  |  | 96616505 | AGC | A | A429 | NA |
| HLA-DRB5 | #4 | 6 | 32487215 | C | CT | R195Q? | NA |
|  |  |  | 32489855 | T | A | D66V | 9.422 |
|  |  |  | 32489874 | T | A | I60F | 0.049 |
|  |  |  | 32489876 | T | A | D59V | 11.35 |
|  | #5 |  | 32487153 | C | G | E216Q | 7.567 |
|  |  |  | 32487158 | G | GCA | T214M? | NA |
|  |  |  | 32487215 | C | CT | R195Q? | NA |
|  |  |  | 32489882 | T | A | H57L | 12.16 |
|  |  |  | 32489888 | A | T | F55Y | 5.704 |
|  |  |  | 32489889 | A | T | F55I | 14.14 |
|  |  |  | 32489892 | G | A | R54W | 8.85 |
|  |  |  | 32489913 | A | G | F47L | 14 |
|  | #6 |  | 32487314 | C | T | R162Q | 12.82 |
|  | #7 |  | 32486438 | G | A | Q220* | 18.08 |
|  |  |  | 32487353 | T | C | N149S | 0.054 |
|  |  |  | 32489855 | T | A | D66V | 9.422 |
|  |  |  | 32489882 | T | A | H57L | 12.16 |
|  |  |  | 32489883 | G | C | H57D | 0.023 |
|  |  |  | 32489888 | A | T | F55Y | 5.704 |
|  |  |  | 32489889 | A | T | F55I | 14.14 |
| DYNC2H1 | #1 | 11 | 103052596 | T | A | L2153* | 47 |
|  | #2 |  | 102995846 | G | A | R560Q | 13.6 |
|  |  |  | 103027318 | C | CTGATTGTAA | P1316LIVT | NA |
|  | #6 |  | 103126213 | GAAGAAGAT | G | EED3433 | NA |
| NEFH | #1 | 22 | 29885638 | T | A | V670E | 0 |
|  | #2 |  | 29885644 | C | A | A672E | 0.001 |
|  | #7 |  | 29885644 | C | A | A672E | 0.001 |
| FASLG | #1 | 1 | 172635091 | G | A | V261I | 14.83 |
|  |  |  | 172635097 | G | C | E263Q | 8.329 |
|  |  |  | 172635109 | G | A | V267I | 12.67 |
|  |  |  | 172635127 | C | A | Q273K | 0.888 |
|  | #3 |  | 172634812 | ATTGT | A | IV168 | NA |
|  |  |  | 172634817 | C | CCGCT | L170RS? | 0.334 |
|  |  |  | 172634821 | C | A | L171I | 0.334 |
|  |  |  | 172635109 | G | A | V267I | 12.67 |
|  |  |  | 172635127 | C | A | Q273K | 0.888 |
|  | #4 |  | 172634812 | ATTGT | A | IV168 | NA |
|  |  |  | 172634817 | C | CCGCT | L170RS? | NA |
|  |  |  | 172634821 | C | A | L171I | 0.334 |
| MUC5B | #1 | 11 | 1260248 | G | A | D1152N | 5.604 |
|  |  |  | 1260249 | A | G | D1152G | 3.325 |
|  |  |  | 1260252 | C | T | T1153I | 8.179 |
|  | #3 |  | 1260248 | G | A | D1152N | 5.604 |
|  |  |  | 1260249 | A | G | D1152G | 3.325 |
| PRSS3 | #1 | 9 | 33794797 | TGA | T | MR3 | NA |
|  | #4 |  | 33794797 | TGA | T | MR3 | NA |
|  |  |  | 33794809 | G | A | S7N | 0.008 |
|  |  |  | 33794812 | G | T | G8V | 0.033 |
|  |  |  | 33796644 | C | G | A72G | 14.93 |
|  |  |  | 33796649 | C | T | P74S | 11.42 |
|  |  |  | 33796686 | C | A | T86N | 8.398 |
|  |  |  | 33796703 | C | G | L92V | 0.246 |
|  |  |  | 33796746 | G | T | G106V | 13.8 |
|  |  |  | 33796758 | T | A | I110N | 13.82 |
|  |  |  | 33796762 | C | G | S111R | 5.004 |
|  |  |  | 33796766 | C | T | Q113* | 7.996 |
|  | #5 |  | 33794797 | TGA | T | MR3 | NA |
|  |  |  | 33794809 | G | A | S7N | 0.008 |
|  |  |  | 33794812 | G | T | G8V | 0.033 |
| HYDIN | #1 | 16 | 70841941 | CTGCG | C | HA4968 | NA |
|  | #4 |  | 70902609 | C | T | R3725Q | 7.379 |
|  | #6 |  | 70852376 | G | T | P4843T | 12.98 |
| MGA | #1 | 15 | 42054444 | G | A | R2543K | 18.68 |
|  | #4 |  | 41999929 | G | T | G731V | 25.7 |
|  | #6 |  | 41961989 | G | GT | S300F? | NA |
| HNRNPCL1 | #3 | 1 | 12907518 | T | A | S209C | 8.642 |
|  |  |  | 12907519 | C | A | Q208H | 7.204 |
|  | #4 |  | 12907284 | T | C | T287A | 0.001 |
|  |  |  | 12907285 | G | C | S286R | 0.23 |
|  | #7 |  | 12907446 | C | T | E233K | 6.762 |
|  |  |  | 12907457 | A | G | M229T | 0 |
|  |  |  | 12907458 | T | A | M229L | 0.004 |
|  |  |  | 12907469 | C | G | S225T | 4.529 |
|  |  |  | 12907508 | T | C | E212G | 0.001 |
|  |  |  | 12907518 | T | A | S209C | 8.642 |
|  |  |  | 12907519 | C | A | Q208H | 7.204 |
| TAS2R46 | #3 | 12 | 11214509 | C | T | V129I | 0.007 |
|  | #5 |  | 11214299 | T | C | I199V | 6.334 |
|  | #7 |  | 11214264 | C | G | Q210H | 6.436 |
| USH2A | #4 | 1 | 216373317 | T | G | S1155R | 15.81 |
|  | #5 |  | 216052233 | G | A | P2811S | 20.1 |
|  | #6 |  | 216591996 | C | A | G171W | 19.45 |
| TAS2R43 | #5 | 12 | 11244027 | T | C | R268G | 0.003 |
|  |  |  | 11244036 | T | G | K265Q | 0.003 |
|  |  |  | 11244067 | A | ATT | S254R? | NA |
|  |  |  | 11244070 | TCC | T | G253 | NA |
|  |  |  | 11244091 | C | T | M246I | 0.004 |
|  |  |  | 11244096 | T | C | I245V | 1.152 |
|  |  |  | 11244102 | G | C | L243V | 2.621 |
|  |  |  | 11244721 | G | C | F36L | 5.223 |
|  |  |  | 11244723 | A | C | F36V | 0.001 |
|  |  |  | 11244731 | A | G | I33T | 0.721 |
|  |  |  | 11244797 | C | A | S11I | 0 |
|  | #6 |  | 11244721 | G | C | F36L | 5.223 |
|  |  |  | 11244723 | A | C | F36V | 0.001 |
|  |  |  | 11244731 | A | G | I33T | 0.721 |
|  | #7 |  | 11244199 | C | G | Q210H | 3.385 |
|  |  |  | 11244721 | G | C | F36L | 5.223 |
|  |  |  | 11244723 | A | C | F36V | 0.001 |
|  |  |  | 11244731 | A | G | I33T | 0.721 |
| GPR98 | #1 | 5 | 90050870 | G | C | L3816F | 12.83 |
|  | #2 |  | 89933756 | C | CT | S744S? | NA |
|  |  |  | 89933758 | C | CTAAAATATATGTTCAGAAATTA | L745LKYMFRNY? | NA |
| ZNF679 | #1 | 7 | 63721279 | G | C | K78N | 5.195 |
|  | #2 |  | 63721287 | A | G | E81G | 5.972 |
| ROBO2 | #1 | 3 | 75986717 | G | A | V25M | 3.198 |
|  | #2 |  | 77600125 | C | CAATATTCA | L406QYS? | NA |
|  |  |  | 77666794 | G | T | A1142S | 21 |
| RNMT | #1 | 18 | 13731869 | C | CAAACTCTTT | S118SNSF | NA |
|  | #2 |  | 13731871 | A | AACTCTTTTCT | T119NSFL? | NA |
|  |  |  | 13737126 | T | TC | V224V? | NA |
| C4BPB | #1 | 1 | 207268780 | G | A | S175N | 0.247 |
|  | #2 |  | 207268780 | G | A | S175N | 0.247 |
|  |  |  | 207268786 | A | C | H177P | 2.195 |
| TEX15 | #1 | 8 | 30702149 | C | T | W1462* | 39 |
|  | #2 |  | 30694422 | C | CTTTATTGAGTTGTTA | R2743RNNSIK | NA |
| DOCK2 | #1 | 5 | 169494534 | G | C | E1496D | 16.78 |
|  | #3 |  | 169507234 | C | T | A1745V | 6.065 |
| ADAMTS2 | #1 | 5 | 178585847 | G | C | Q337E | 28.6 |
|  | #4 |  | 178548685 | G | T | S1052* | 40 |
| INVS | #1 | 9 | 103054721 | G | A | E728K | 17.01 |
|  | #4 |  | 103046637 | A | G | E607G | 13.24 |
| ZNF880 | #1 | 19 | 52877732 | A | G | K107R | NA |
|  | #4 |  | 52888074 | G | GAT | G414G? | NA |
| CUL7 | #1 | 6 | 43020271 | C | T | G138S | 22 |
|  | #4 |  | 43010692 | C | T | D1249N | 17.54 |
| NHSL2 | #1 | X | 71360567 | C | G | Q1057E | 0.034 |
|  | #4 |  | 71360022 | C | T | P875L | 18.31 |
| CD55 | #1 | 1 | 207520819 | A | C | D370A | 1.415 |
|  | #5 |  | 207523493 | G | A | M364I | 3.954 |
| USP24 | #1 | 1 | 55622999 | C | A | K424N | 14.69 |
|  | #5 |  | 55620115 | C | T | V527I | 16.94 |
| VPS13A | #1 | 9 | 79934573 | G | GTTTTTTA | W1800CFL? | NA |
|  | #5 |  | 79968357 | GAC | G | T2485 | NA |
| IL33 | #1 | 9 | 6241703 | TAAA | T | K4 | NA |
|  | #5 |  | 6256046 | G | A | E231K | 25.9 |
| ADAMTS12 | #1 | 5 | 33614463 | T | C | N803D | 27.8 |
|  | #6 |  | 33576452 | TG | T | P1226 | NA |
| TAS2R31 | #1 | 12 | 11183837 | A | G | I33T | 5.251 |
|  | #6 |  | 11183697 | C | A | V80L | 2.153 |
|  |  |  | 11183708 | T | C | Y76C | 5.935 |
|  |  |  | 11183722 | A | C | F71L | 0.01 |
| TPSB2 | #1 | 16 | 1279732 | C | A | G23V | 12.04 |
|  | #6 |  | 1279714 | A | G | V29A | 8.603 |
|  |  |  | 1279717 | C | T | R28Q | 9.729 |
| MUC12 | #1 | 7 | 100616310 | G | C | E91D | 2.375 |
|  |  |  | 100616313 | T | A | S92R | 2.387 |
|  |  |  | 100616320 | G | A | V95M | 3.904 |
|  |  |  | 100616336 | G | A | W100* | 11.98 |
|  |  |  | 100616338 | A | G | M101V | 0.417 |
|  |  |  | 100616344 | G | A | G103R | 2.979 |
|  | #7 |  | 100616227 | A | T | R64W | 3.244 |
|  |  |  | 100616229 | G | C | R64S | 2.205 |
| OR2T4 | #1 | 1 | 248525100 | G | A | G73E | 8.963 |
|  |  |  | 248525135 | G | A | A85T | 4.912 |
|  |  |  | 248525138 | C | T | H86Y | 4.27 |
|  | #7 |  | 248525100 | G | A | G73E | 8.963 |
| CNN2 | #1 | 19 | 1037756 | G | A | G284S | 28.7 |
|  |  |  | 1037766 | G | A | R287Q | 19.93 |
|  | #7 |  | 1037766 | G | A | R287Q | 19.93 |
| CNKSR3 | #2 | 6 | 154743741 | G | T | P282T | 20.4 |
|  | #3 |  | 154732140 | C | A | D403Y | 15.56 |
| LAMA3 | #2 | 18 | 21511146 | G | T | V2853L | 14.59 |
|  | #3 |  | 21453105 | C | A | Q33K | 24.1 |
| EML6 | #2 | 2 | 55056611 | A | ATGCTAAAGATTCC | K282MLKIP? | NA |
|  | #3 |  | 55040448 | T | A | Y93N | 21.6 |
| TTN | #2 | 2 | 179556813 | G | GTGTTTTTACTGTTT | P10564PNSKN? | NA |
|  |  |  | 179611064 | C | A | V5355L | 6.771 |
|  |  |  | 179642503 | C | A | E1470* | 46 |
|  | #4 |  | 179428105 | G | T | S27585Y | 14.93 |
| COL4A5 | #2 | X | 107868992 | T | A | L1025H | 10.48 |
|  | #5 |  | 107834817 | C | G | P456A | 7.616 |
| KNDC1 | #2 | 10 | 134980924 | C | T | R48C | 11.02 |
|  | #5 |  | 135012509 | C | T | R833* | 40 |
| EPB41 | #2 | 1 | 29319962 | T | A | L197I | 11.9 |
|  |  |  | 29319963 | T | A | L197* | 15.21 |
|  | #5 |  | 29438905 | C | T | T814I | 28.4 |
| HLA-B | #2 | 6 | 31324144 | T | G | Y140S | 0.011 |
|  | #5 |  | 31324552 | G | C | R86G | 6.275 |
| PKHD1L1 | #2 | 8 | 110412514 | A | ACC | I408T? | NA |
|  | #6 |  | 110453081 | G | A | G1367R | 20.7 |
| KLHL23 | #2 | 2 | 170591981 | G | GAGAGTTTTATTTTATTTTT | E153ESFILFL? | NA |
|  | #7 |  | 170591937 | A | G | N138S | 20.8 |
| EVPL | #3 | 17 | 74011444 | G | A | A653V | 19.65 |
|  |  |  | 74011445 | C | A | A653S | 34 |
|  | #4 |  | 74017617 | ACTCCATCTTCAGGGCCTC | A | EALKME309 | NA |
| FZR1 | #3 | 19 | 3526379 | T | TATTCCC | F128YSL | NA |
|  | #4 |  | 3526374 | G | GGC | G126G? | NA |
|  |  |  | 3526377 | T | TTTAC | L127LY? | NA |
|  |  |  | 3526379 | T | TATTCCC | F128YSL | NA |
| CACNA2D1 | #3 | 7 | 81599215 | T | A | T776S | 11.77 |
|  | #5 |  | 81799909 | G | A | A104V | 28.1 |
| CDC27 | #3 | 17 | 45234406 | CA | C | A238 | NA |
|  |  |  | 45234417 | A | G | I235T | 16.6 |
|  | #5 |  | 45214690 | G | A | R587W | 21.7 |
| KCNT2 | #3 | 1 | 196309693 | C | T | V521I | 16.76 |
|  | #6 |  | 196274404 | G | T | A852D | 23.7 |
| KIAA1468 | #3 | 18 | 59931309 | T | G | L813R | 17.37 |
|  | #6 |  | 59895774 | G | A | G464E | 0.011 |
| EMP2 | #3 | 16 | 10626861 | T | TTG | R135S? | NA |
|  |  |  | 10631869 | C | A | A78S | 5.93 |
|  |  |  | 10631877 | C | G | C75S | 5.486 |
|  | #7 |  | 10631877 | C | G | C75S | 5.486 |
| OR4C5 | #4 | 11 | 48387285 | A | AGTCTTTAGTAG | C244CLLK? | NA |
|  | #5 |  | 48387155 | T | C | Y288C | 2.41 |
|  |  |  | 48387930 | T | C | I30V | 0.004 |
|  |  |  | 48387934 | G | T | N28K | 6.371 |
|  |  |  | 48387939 | C | G | V27L | 9.711 |
|  |  |  | 48387945 | C | CCA | F24F? | NA |
|  |  |  | 48387946 | G | T | F24L | 2.36 |
| MST1 | #4 | 3 | 49723321 | AGCGCTG | A | QR406 | NA |
|  | #5 |  | 49723321 | AGCGCTG | A | QR406 | NA |
| ARHGAP32 | #4 | 11 | 128963571 | A | T | M145K | 12.46 |
|  |  |  | 128963569 | TC | T | M145 | NA |
|  | #5 |  | 128840090 | G | A | S1659F | 16.15 |
| NLGN4X | #4 | X | 5811532 | G | A | L613F | 15.4 |
|  | #5 |  | 5811532 | G | A | L613F | 15.4 |
| ZNF611 | #4 | 19 | 53208749 | C | G | S520T | 1.295 |
|  | #6 |  | 53208521 | C | G | C596S | 15.97 |
| HLA-DQA1 | #4 | 6 | 32609207 | C | T | A68V | 0.008 |
|  |  |  | 32609254 | G | T | G84C | 9.414 |
|  |  |  | 32609255 | G | T | G84V | 7.745 |
|  |  |  | 32609873 | G | C | Q152H | 0.005 |
|  | #7 |  | 32609254 | G | T | G84C | 9.414 |
|  |  |  | 32609255 | G | T | G84V | 7.745 |
| HERC2 | #5 | 15 | 28518112 | C | A | S280I | 13.15 |
|  |  |  | 28518114 | TC | T | G279 | NA |
|  |  |  | 28518130 | G | C | A274G | 2.432 |
|  | #6 |  | 28518114 | TC | T | G279 | NA |
|  |  |  | 28518130 | G | C | A274G | 2.432 |
|  |  |  | 28518136 | G | A | T272M | 7.967 |
| ASTN2 | #6 | 9 | 119903716 | G | A | Q353* | 38 |
|  | #7 |  | 119903623 | G | A | R384W | 14.32 |
| MROH2B | #6 | 5 | 41067220 | C | T | R64K | 21.9 |
|  | #7 |  | 41061811 | C | T | S159N | 19.96 |
| SP8 | #6 | 7 | 20824778 | C | A | V220L | 12.45 |
|  | #7 |  | 20825145 | GGCAGCCGCGGCTGCTGCCGCGGCCGCC | G | AAAAAAAAAA88A | NA |
| PRSS1 | #6 | 7 | 142460764 | G | A | V227I | 0 |
|  | #7 |  | 142458526 | A | G | N54S | 0.009 |
|  |  |  | 142458527 | C | G | N54K | 9.055 |
|  |  |  | 142458531 | C | T | Q56* | 13.15 |

chrom, chromosome; pos, bp location; ref/alt, reference and altered nucleotides at mutated position; AA change, amino acid change; CADD, Combined Annotation Dependent Depletion score; CADD score ≥10 indicates a position within the top 10% most deleterious mutations. A score of ≥20 indicates a position within the top 1% most deleterious mutations.
