# Supplementary figures and images for: Overall survival in EGFR mutated non-small-cell lung cancer patients treated with afatinib after EGFR TKI and resistant mechanisms upon disease progression
Source: PLoS One. 2017 Aug 30;12(8):e0182885. doi: 10.1371/journal.pone.0182885 (PMC5576694; doi:10.1371/journal.pone.0182885)

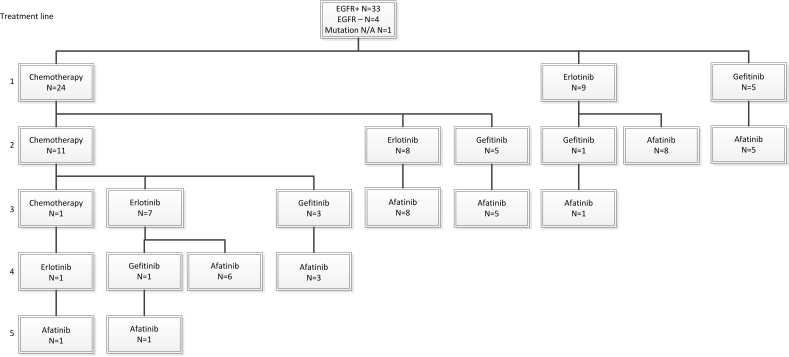

Supplement: S1 Fig — Organogram of 38 treated patients where afatinib is given in different treatment lines (1–5). Chemotherapy was variable, e.g. cisplatinum/pemetrexed, carboplatinum/paclitaxel/bevacizumab, docetaxel, pemetrexed. (TIF) [file pone.0182885.s002.tif]

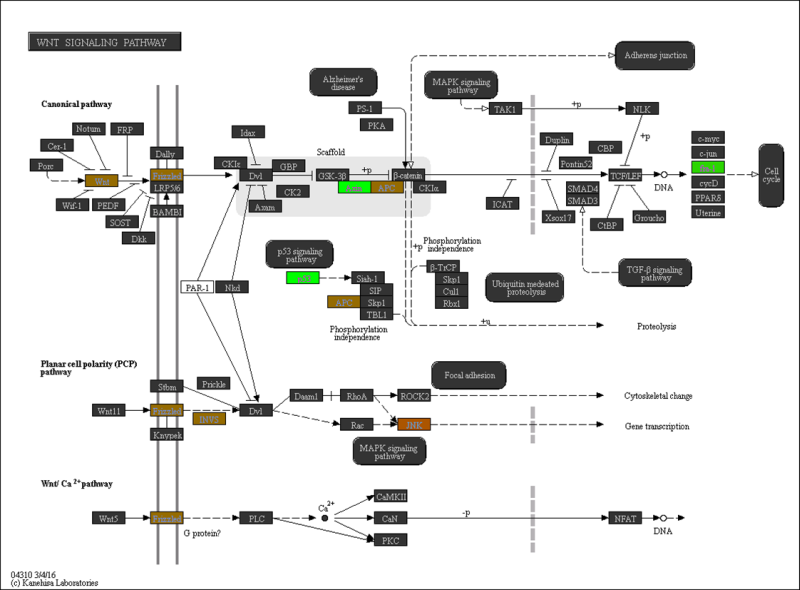

Supplement: S2 Fig — Color boxes are the different mutations found in different samples. Multiple mutated genes involved in the Wnt pathway are shown. (TIF) [file pone.0182885.s003.tif]

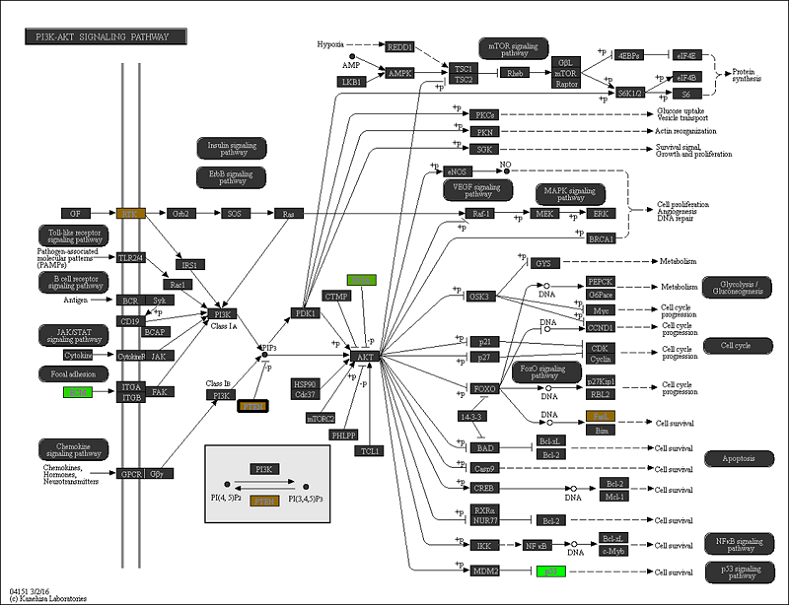

Supplement: S3 Fig — Color boxes are the different mutations found in different samples. (TIF) [file pone.0182885.s004.tif]

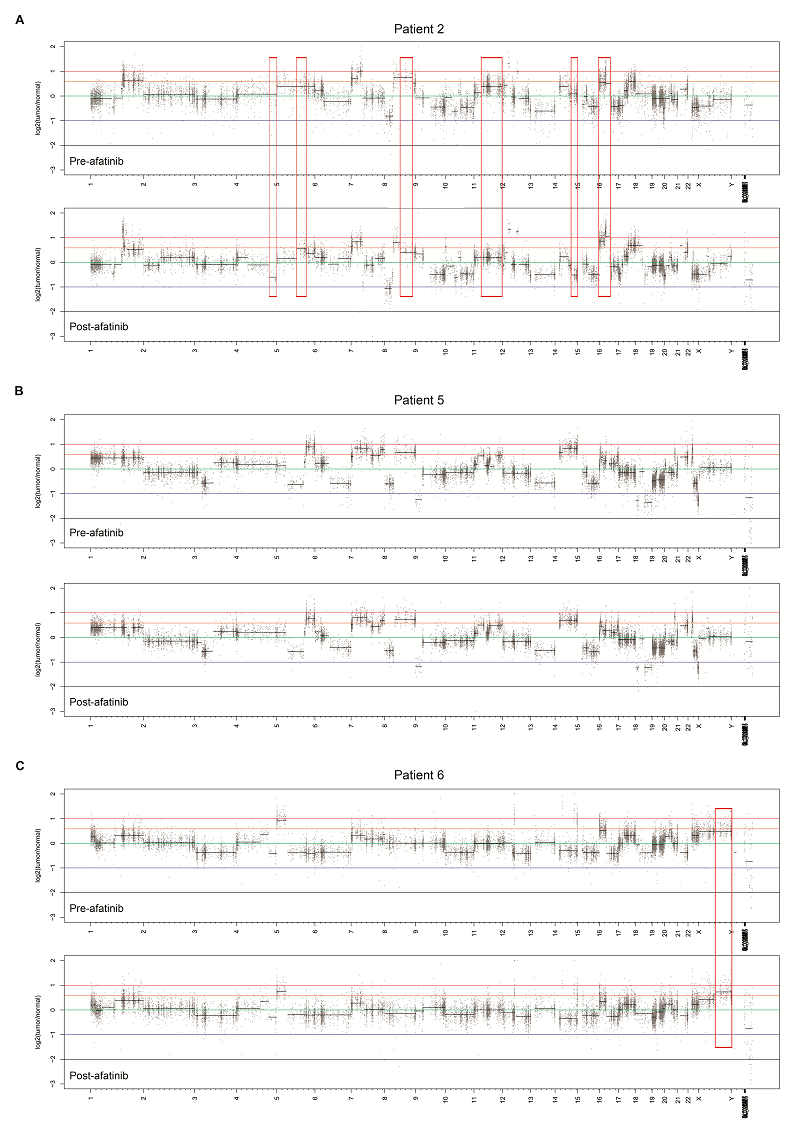

Supplement: S4 Fig — Overview of the copy numbers and allele frequencies of pre-afatinib (top) and post-afatinib biopsies (bottom) in patients 2 (A), 5 (B) and 6 (C). The boxes indicate aberrations between pre-afatinib and post-afatinib biopsies with either copy number gain or loss. (TIF) [file pone.0182885.s005.tif]
